# Video and Infographic Messages From Primary Care Physicians and Influenza Vaccination Rates: A Randomized Clinical Trial

**DOI:** 10.1001/jamanetworkopen.2025.26514

**Published:** 2025-08-13

**Authors:** Peter G. Szilagyi, Emma J. Clark, O. Kenrik Duru, Alejandra Casillas, Michael K. Ong, Sitaram Vangala, Chi-Hong Tseng, Christina Albertin, Sharon G. Humiston, Mindy K. Ross, Sharon Evans, Arjun Kumar, Ilona Chakarian, Michael Sloyan, Craig R. Fox, Cynthia M. Rand, Carlos Lerner

**Affiliations:** 1 Department of Pediatrics, UCLA Mattel Children’s Hospital, University of California, Los Angeles; 2 Department of Medicine, David Geffen School of Medicine, University of California, Los Angeles; 3 Department of Health Policy and Management, Fielding School of Public Health, University of California, Los Angeles; 4 VA Greater Los Angeles Healthcare System, Los Angeles, California; 5 Department of Medicine Statistics Core, David Geffen School of Medicine, University of California, Los Angeles; 6 Immunize.org, St Paul, Minnesota; 7 Department of Information Services and Solutions, UCLA Health System, Los Angeles, California; 8 Anderson School of Management, University of California, Los Angeles; 9 Department of Psychology, University of California, Los Angeles; 10 Department of Pediatrics, University of Rochester Medical Center, Rochester, New York

## Abstract

**Importance:**

Influenza vaccination coverage in the US is low, due largely to vaccine hesitancy. Personalized messages from trusted physicians may raise vaccination rates.

**Objective:**

To evaluate the effects of physician-created videos and infographics containing physician photographs on influenza vaccination rates.

**Design, Setting, and Participants:**

This 3-arm randomized clinical trial included patients from 6 months to older than 65 years of age served by 21 primary care physicians in 21 practices at UCLA Health who were listed in the electronic health record as not receiving influenza vaccination between October 31, 2023, and April 1, 2024.

**Intervention:**

Patients within each physician patient population were randomized to receive usual care or a physician-created video or an infographic with their physician’s photograph encouraging receipt of influenza vaccinations. Primary care physicians used a suggested script to create a brief video with their smartphone or a video conference recording, or an infographic containing their photograph and a similar script was created, both encouraging patients to receive influenza vaccinations. The video or infographic was sent via the patient portal up to 3 times (October, November, and December).

**Main Outcomes and Measures:**

All analyses used the evaluable patient population. The primary outcome was end-of-season influenza vaccination rates by Aril 1, 2024. A secondary outcome was timely vaccination by December 31, 2023.

**Results:**

Among 22 233 patients, 3200 were children younger than 18 years (14.4%), 14 704 were adults 18 to 64 years of age (66.1%), and 4329 were adults 65 years or older (19.5%); 13 973 (62.8%) were female. Overall, 18 878 patients (84.9%) had private insurance. The rates of influenza vaccination across the entire study population by April 1, 2024, were 3479 of 7417 (46.9%) for usual care, 3557 of 7410 (48.0%) for the video arm, and 3518 of 7406 (47.5%) for the infographic arm. Neither intervention had significantly higher rates of vaccination than usual care (adjusted risk ratio, 1.03 [95% CI, 1.00-1.06] for the video group; adjusted risk ratio, 1.02 [95% CI, 0.99-1.06] for the infographic group). In the post hoc analysis, children in both intervention arms had higher vaccination rates by April 1, 2024: 618 of 1058 children in the video group (58.4%) (*P* < .001) and 598 of 1085 children in the infographics group (55.1%), each compared with 576 of 1057 (54.5%) in the usual care group (*P* = .04).

**Conclusions and Relevance:**

In this randomized clinical trial, neither physician-created videos nor infographics increased influenza overall vaccination rates by April 1, 2024. However, among children, both interventions were effective by end of influenza season. Messages personalized by physicians may represent a potential strategy to improve influenza vaccination among children, but further innovations are needed across the entire age spectrum.

**Trial Registration:**

ClinicalTrials.gov Identifier: NCT06062264

## Key Points

**Question:**

Can a patient portal message with either a physician-created video or an infographic with a physician photograph increase end-of-season influenza vaccination rates?

**Findings:**

In this randomized clinical trial of 22 233 patients in UCLA Health, neither physician-created video messages nor infographic messages containing the physician’s photograph increased end-of-season influenza vaccination rates overall compared with usual care. By contrast, both interventions, especially video messages, increased rates among children.

**Meaning:**

Findings from this study indicate that health systems should consider physician-created video messages to increase child influenza vaccination rates and that further investigation of innovations for improving influenza vaccination rates across the entire population is warranted.

## Teaser

This randomized clinical trial investigates whether physician-created videos or infographics containing physician photographs delivered via a patient portal affect influenza vaccination rates compared with usual care.

## Introduction

Influenza infection causes substantial morbidity and mortality in the US.^1,2^ A US Healthy People 2030 goal is to achieve 70% vaccination coverage, yet vaccination rates have plateaued since 2010^3^ and declined since 2020. For the 2023-2024 influenza vaccination season, coverage was 55.4% for children 6 months through 17 years of age, and 32.8% for adults 18 to 49 years, 46.2% for adults 50 to 64 years, and 69.7% for adults 65 years or older.^4^

A major cause of low influenza vaccination is patient vaccine hesitancy. While most people trust their physicians overall,^5,6^ many don’t feel that the benefits of an influenza vaccine outweigh its safety and believe that they are not at risk from influenza disease.^7,8^ Vaccine hesitancy may be increasing since the COVID-19 pandemic.^9^ Strategies built on trusted messengers,^10^ such as physicians,^11,12^ are needed.

One evidence-based strategy for vaccinations, recommended by the Guide to Community Preventive Services and other experts, is patient reminders or communications sent by health practices, health systems, or public health^13,14,15^ by telephone, autodialers, patient portals,^16^ or text messages.^17,18,19,20,21,22,23,24,25^ While some studies have found that patient reminders can increase influenza vaccination coverage (particularly if sent just before scheduled appointments),^23,26^ the effectiveness of routine patient reminders has appeared to wane, with many studies finding no or a small effect.^23,27,28^

Between 2017 and 2024, several randomized clinical trials (RCTs) evaluated the effect of messages sent via the electronic health record (EHR) patient portal or text on influenza vaccination rates across a large health system.^29^ We tested types of messages (monthly, tailored to age or diabetes, and positive or negative framing),^30^ modalities (portal, text message),^26^ and access strategies (self-scheduling appointments,^31^ preappointment reminders).^26^ Portal messages included the name of the primary care physician (PCP). Except for text messages sent before scheduled appointments^26^ and reminders for second influenza vaccinations for eligible young children,^32^ these reminders did not increase end of season vaccination coverage at the population level, likely due to patient vaccine hesitancy.

While studies have assessed the impact of physician messages used as public service announcements,^33,34^ to our knowledge, video messages sent to patients by their physicians to improve preventive services have not been studied. Here we report the results of a 3-arm RCT to evaluate the effect of 2 interventions: (1) brief videos filmed by PCPs and based on prewritten scripts encouraging their patients to receive an influenza vaccine, and (2) an infographic created by our research team that contained information similar to that in the videos plus a photograph of the patient’s PCP for personalization. We randomized patients, within each PCP’s list of patients, to usual care, a video, or an infographic, hypothesizing that each intervention would increase influenza vaccination rates, with videos being most effective.

## Methods

### Study Design

**Figure 1.**
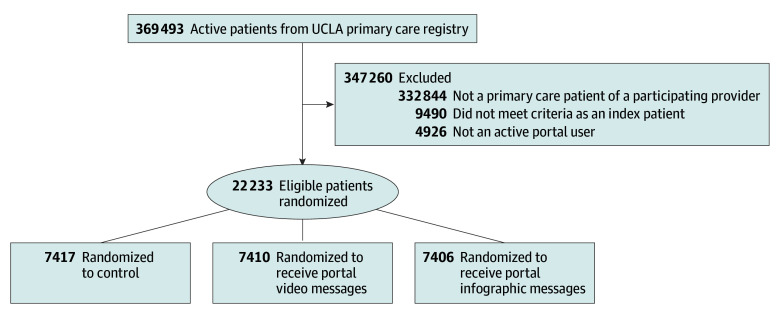
Consolidated Standards of Reporting Trials (CONSORT) Diagram UCLA indicates University of California, Los Angeles.

This RCT was approved by the University of California, Los Angeles (UCLA), Institutional Review Board with a patient consent waiver because the interventions focused on a universally recommended vaccination, and the risk from the intervention was minimal. We conducted a 3-arm RCT (Figure 1) between October 3, 2023, and April 1, 2024. We randomly allocated UCLA Health patients of 21 PCPs (internal medicine, medicine-pediatrics, family medicine, or pediatrics) to (1) usual care, (2) video message self-recorded by physicians, sent by the health system to patients via the patient portal, or (3) infographics sent by the health system via the portal. Results are reported per the Consolidated Standards of Reporting Trials (CONSORT) reporting guideline. The trial protocol is provided in Supplement 1.

### Study Participants

The unit of randomization was patients. Statisticians (S.V. and C.-H.T.) randomly selected 27 practices of 79 primary care internal medicine, medicine-pediatrics, and family medicine practices; 23 practices agreed to participate. We contacted physicians in these practices up to 3 times until we accrued 21 physicians from 21 practices who agreed to have their patients randomized and to record a video for their own patients who were randomized to the video study arm.

UCLA Health attributed patients to PCPs who had at least 2 PCP visits within 3 years, or at least 1 PCP visit with a preventive service code within 1 year. Other eligibility criteria as a primary care patient of a participating physician included being older than 6 months of age and being an active portal user (>1 login across the last 12 months, not including the initial account login). Patients were ineligible if they were not a primary care patient of a participating physician or not an active patient portal user. As before,^26,29,30,31,32^ we selected 1 patient per family, identifying family units by matching addresses, insurance member numbers, phone numbers, and patient guarantors’ identifiers. Statisticians (S.V. and C.-H.T.) randomly allocated an index patient from each family to a study arm; other researchers and their physicians were blinded to allocation. Other family members received usual care and were excluded from analyses. Patients from prior studies by our team were included if selected as index patients.

#### Interventions: Physician Videos and Infographic Messages Sent by Patient Portal

UCLA Health uses its EHR (Epic Systems) to send portal messages to patients. Reminder portal messages were sent to patients who, according to the EHR incorporating both UCLA and available external data, were still due influenza vaccination. Both video and infographic group participants were notified by email from the portal that they had a message from their physician, with a subject line including the physician’s name (eg, “Video message from [PCP name] regarding your flu vaccine”) (eAppendix 1 in Supplement 2). Messages for both intervention groups had a link routing patients to their patient portal login, which then had another link routing them to a questionnaire with either the video link or the infographic (the questionnaire was the technically available method to deliver these messages). Messages were in English (98% of patients listed English as their preferred language for the portal) and below seventh grade reading level by Flesch-Kincaid analysis. Several UCLA patients reviewed draft messages for content and construct validity. Clinicians were not copied on messages; patients were not notified about the study.

##### Video Message

Physicians created their own video using their smartphone or via virtual consultation with a research team member (E.J.C.) and a UCLA Health communications staff member who recorded their video using a common videotelephony software program. We provided physicians with general instructions plus an adult sample patient script (for internal medicine, medicine-pediatric, or family medicine physicians [177 words]), or a pediatric script (for pediatricians [191 words]) (eAppendix 2 in Supplement 2), with content based on the literature on messaging.^35,36^ In addition to this core content, we suggested key themes based on findings from prior studies for physicians to consider including in their messages plus other possible phrases or sentences to use.^31,32^ Physicians were encouraged to personalize their messages. Otherwise, instructions were to create a video less than 60 seconds in duration or fewer than 225 words, to use a caring and upbeat tone, and to look directly into the camera.

Physicians who recorded their own videos (16 of 21) emailed their video file to a research team member (E.J.C.). The communications group occasionally optimized the sound and visual quality, added the name of the physician as a clip at the bottom of screen to the video, and uploaded the videos to the UCLA Health YouTube channel as unlisted videos accessible only by the patient. YouTube links were on the portal video messages. Physicians received $50 to record videos.

Video messages had an accompanying questionnaire asking patients if they watched the video and whether and where they planned to obtain the influenza vaccine. Up to 3 portal messages containing the video were sent (in October, November, and December) for patients who remained unvaccinated.

##### Infographic

Based on the literature^37,38^ and our team’s prior experience, we designed a simple infographic (eAppendix 3 in Supplement 2) indicating that “Dr. ‘X’ wants you to get a flu vaccine, I suggest the flu vaccine for my family, the flu vaccine keeps you healthy and safe, please schedule a vaccine visit now, and thanks for trusting me with your care.” The infographic contained a headshot photograph of the physician and a UCLA Health logo. Infographic files were uploaded to a website and linked to portal infographic messages. Patients in the infographic and video message study arms were sent identical questionnaires (the only difference was that subject lines mentioned video message for the video study arm and message for the infographic study arm) and identical numbers of messages on the same schedules.

##### Children

Portal messages (for both intervention arms) for pediatric practices were addressed to the parent of [the child’s first name], except that messages for adolescents older than 12 years with portal privileges were addressed to them. To avoid having medicine-pediatric or family medicine physicians record 2 messages, portal messages for children younger than 12 years in those practices were addressed to the child’s name but sent to the parent guardian.

##### Usual Care Control Group

This group received usual care, with no communication from our research team. UCLA Health sends multiple reminders to their employees about the importance of an influenza vaccination and some patients were UCLA employees.

### Survey of PCPs

After April 1, 2023, we sent an email survey via Research Electronic Data Capture (REDCap) (up to 5 follow-up surveys for nonresponders) to the 21 PCPs about their experience in creating the videos (eAppendix 4 in Supplement 2). We asked about the modality physicians used to record the video, the feasibility of creating a video (how long it took, how easy it was), how closely they followed the script vs creating their own script, their confidence in recording the video, patient responses to the videos, and whether they would do it again. These questions were adapted from the System Usability Scale^39^ and pilot tested among the research group for content validity. Physicians received $50 after completing the survey.

### Measures

#### Patient Characteristics

We assessed patient age, sex, insurance, race and ethnicity, and prior influenza vaccination in 2021-2022 and 2022-2023 because these factors have been associated with influenza vaccination rates.^3,4^ Race and ethnicity were obtained from the EHR based on patient self-identification and were classified by study statisticians (S.V. and C.-H.T.) as Asian, Black, White, or other, which included unknown or racially diverse, and Hispanic or non-Hispanic ethnicity.

#### Influenza Vaccination Data

The UCLA Health EHR automatically incorporates influenza vaccine dates and site of vaccination from any UCLA site, the California Immunization Registry (CAIR), Surescripts pharmacy benefits manager, and Care Everywhere (other Epic Systems sites). We integrated all external data sources before analysis by having the UCLA Health EHR team perform an extra search.

### Outcome Measures

The primary outcome was receipt of any influenza vaccination for the total study population received during the 2023-2024 season (between October 3, 2023, and April 1, 2024), as noted in the EHR. The prespecified secondary analyses was receipt of any influenza vaccination among pediatric patients. Secondary outcomes included (1) other subgroup analyses, including solely pediatric patients; (2) influenza vaccination by December 31, 2023 (referred to as timely vaccination since influenza infections can appear by January^2^); and (3) rates at which patients opened the portal messages.

### Power Calculation

We conducted a power calculation assuming a mean of 50% influenza vaccination rates (most conservative estimate), 20 physicians, and a mean of 1000 patients would provide 80% power to detect a 2.7 percentage-point improvement in either video or infographic intervention arm vs the control arm. This calculation assumed a 2.5% significance level (2-fold Bonferroni correction) and a χ^2^ test.

### Statistical Analysis

All analyses used the evaluable population. Our primary analyses compared vaccination rates between study arms using mixed-effects Poisson regression with robust SEs. Models included main effects for modality (video vs infographic vs control), plus random physician effects, and controls for patient characteristics (age, sex, race and ethnicity, insurance, and prior influenza vaccination). Secondary subgroup analyses were performed by fitting separate models, including interactions between each subgrouping factor of interest and each of the main intervention effects, and performing appropriate linear contrasts. Sensitivity analyses using mixed-effects logistic regression and Poisson regression with physician fixed effects were performed. A secondary post hoc analysis evaluating the outcome of timely vaccination by December 31, 2023, was performed using the same specification as the primary analysis. For the primary outcome, a 2-sided .025 significance level was used (2-fold Bonferroni correction for comparison of each intervention to control). All other analyses used a 2-sided *P* < .05 for statistical significance. Analyses were performed using SAS, version 9.4 (SAS Institute Inc).

## Results

### Practice and Patient Characteristics

A total of 22 233 patients were randomized. The groups were well balanced by age, sex, primary insurer, race and ethnicity, and influenza vaccination in the prior 2 years (Table 1). Of 22 233 participants, 2414 (10.8%) identified as Asian, 1016 (4.6%) as Black, 11 050 (49.7%) as White, and 7753 (34.9%) as racially diverse. Overall, 13 973 (62.8%) were female, and 8260 (37.2%) were male. Patients comprised 3200 children younger than 18 years (14.4%), 14 704 adults 18 to 64 years of age (66.1%), and 4329 adults 65 years or older (19.5%). In total, 18 878 (84.9%) had private insurance. No patient concerns were received.

**Table 1.** Characteristics of the Sample by Study Group

Patient characteristic	Patients, No. (%)		
	Control (n = 7417)	Video message (n = 7410)	Infographic message (n = 7406)
Age group, y			
0.5 (6 mo) to <18	1057 (14.3)	1058 (14.3)	1085 (14.7)
18-49	3344 (45.1)	3362 (45.4)	3416 (46.1)
50-64	1537 (20.7)	1546 (20.9)	1499 (20.2)
≥65	1479 (19.9)	1444 (19.5)	1406 (19.0)
Sex			
Female	4668 (62.9)	4647 (62.7)	4658 (62.9)
Male	2749 (37.1)	2763 (37.3)	2748 (37.1)
Primary insurer			
Private	6269 (84.5)	6307 (85.1)	6302 (85.1)
Public	1035 (14.0)	996 (13.4)	996 (13.4)
Other, unknown, or multiple	113 (1.5)	107 (1.4)	108 (1.5)
Race^a^			
Asian	843 (11.4)	776 (10.5)	795 (10.7)
Black	338 (4.6)	326 (4.4)	352 (4.8)
White	3635 (49.0)	3797 (51.2)	3618 (48.9)
Other (unknown or racially diverse)^b^	2601 (35.1)	2511 (33.9)	2641 (35.7)
Ethnicity^a^			
Hispanic	1044 (14.1)	1020 (13.8)	1008 (13.6)
Non-Hispanic or unknown	6373 (85.9)	6390 (86.2)	6398 (86.4)
Influenza vaccine history (past 2 y)			
None	1964 (26.5)	1950 (26.3)	1974 (26.7)
Prior vaccination	5453 (73.5)	5469 (73.8)	5432 (73.3)

^a^ Race and ethnicity were obtained from the electronic health record based on patient self-identification and were classified by study statisticians (S.V. and C.-H.T.) as Asian, Black, White, or other, which included unknown or racially diverse, and Hispanic or non-Hispanic ethnicity.

^b^ Participants could choose more than 1 race when providing data about race at patient care visits.

### End of Season Influenza Vaccination (Primary Outcome)

Overall and across all ages (Table 2), 3479 of 7417 (46.9%) patients in the control arm, 3557 of 7410 (48.0%) patients in the video arm, and 3518 of 7406 (47.5%) patients in the infographic arm received an influenza vaccination by April 1, 2024. Neither intervention arm had statistically higher vaccination rates than the control arm (*P* = .06 for video vs control; *P* = .16 for infographic vs control). Table 3 shows adjusted risk ratios (ARRs) and 95% CIs for receipt of influenza vaccination by April 1, 2024, by study arm (video or infographic vs control). The likelihood of vaccination in the video group was not significantly different from patients in the control group (ARR, 1.03 [95% CI, 1.00-1.06]) or the infographic group (ARR, 1.02 [95% CI, 0.99-1.06]).

**Table 2.** Vaccination Rates Before April 1, 2024, by Study Arm for All Patients and Subgroups

Patient characteristic	Patient vaccination				
	Control, No. (%)	Video Message		Infographic Message	
		No. (%)	*P* value vs control	No. (%)	*P* value vs control
All patients	3479 (46.9)	3557 (48.0)	.06	3518 (47.5)	.16
Age group, y					
0.5 (6 mo) to <18	576 (54.5)	618 (58.4)	<.001	598 (55.1)	.04
18-49	1241 (37.1)	1318 (39.2)	.06	1315 (38.5)	.17
50-64	696 (45.3)	708 (45.8)	.98	699 (46.6)	.98
≥65	961 (65.0)	915 (63.4)	.72	911 (64.8)	.29
Sex					
Female	2189 (46.9)	2226 (47.9)	.006	2208 (47.4)	.23
Male	1284 (46.7)	1332 (48.2)	.88	1314 (47.8)	.47
Primary insurer					
Private	2783 (44.4)	2926 (46.4)	.008	2899 (46.0)	.03
Public	637 (61.5)	579 (58.1)	.59	578 (58.0)	.51
Other or unknown	52 (46.0)	53 (49.5)	.74	47 (43.5)	.44
Race^a^					
Asian	514 (61.0)	452 (58.2)	.43	479 (60.3)	.73
Black	208 (33.1)	105 (32.2)	.43	109 (31.0)	.86
White	1774 (48.8)	1959 (51.6)	.005	1838 (50.8)	.15
Other (racially diverse or unknown)^b^	1074 (41.3)	1045 (41.6)	.89	1096 (41.5)	.63
Ethnicity^a^					
Hispanic	432 (41.4)	434 (42.5)	.70	408 (40.5)	.70
Non-Hispanic or unknown	3040 (47.7)	3125 (48.9)	.049	3116 (48.7)	.12
Influenza vaccine history (past 2 y)					
None	183 (9.3)	211 (10.8)	.02	203 (10.3)	.06
Prior vaccination	3294 (60.4)	3352 (61.3)	.17	3319 (61.1)	.24

^a^ Race and ethnicity were obtained from the electronic health record based on patient self-identification and were classified by study statisticians (S.V. and C.-H.T.) as Asian, Black, White, or other, which included unknown or racially diverse, and Hispanic or non-Hispanic ethnicity.

^b^ Participants could choose more than 1 race when providing data about race at patient care visits.

**Table 3.** ARRs for Influenza Vaccination by April 1, 2024, by Study Group and Patient Characteristics, Using a Mixed Effects Poisson Model (Clustering by Primary Care Practitioner) of Vaccination Status

Group	ARR (95% CI)
Study group modality	
Control	1 [Reference]
Video	1.03 (1.00-1.06)
Infographic	1.02 (0.99-1.06)
Age group, y	
<18	1 [Reference]
18-49	0.74 (0.66-0.82)
50-64	0.87 (0.78-0.97)
≥65	1.09 (0.97-1.22)
Sex	
Male	1.00 (0.97-1.03)
Female	1 [Reference]
Primary insurer	
Private	1 [Reference]
Public	1.00 (0.95-1.05)
Other or unknown	0.98 (0.85-1.12)
Race	
Asian	1.13 (1.10-1.16)
Black	0.84 (0.76-0.92)
White	1 [Reference]
Other (racially diverse or unknown)^a^	0.91 (0.89-0.94)
Ethnicity	
Hispanic	0.96 (0.91-1.02)
Non-Hispanic or unknown	1 [Reference]
Influenza vaccine history (past 2 y)	
Yes	5.59 (4.01-7.77)
No	1 [Reference]

Abbreviation: ARR, adjusted risk ratio.

^a^ Participants could choose more than 1 race when providing data about race at patient care visits.

### Influenza Vaccination by April 1, 2024, for Pediatric Patients (Prespecified Secondary Analysis)

Among children younger than 18 years, those in the video arm (618 of 1058 [58.4%]; *P* < .001) and infographic arm (598 of 1085 [55.1%]; *P* = .04) had significantly higher vaccination rates (Table 2) than the control arm (576 of 1057 [54.5%]). The number needed to treat to achieve 1 additional influenza vaccination among children was 26 in the video arm and was 167 in the infographic arm.

#### Subgroup Analyses (Post Hoc)

Statistically higher vaccination rates were noted for the video group vs the control group among 5 additional subgroups of patients: female, privately insured, White, non-Hispanic, and those without influenza vaccination in the prior 2 years (Table 2). Statistically higher vaccination rates were noted for the infographic group (2899 [46.0%]) vs control group (2783 [44.4%]) among privately insured patients (*P* = .03), although the findings for patients with no vaccination in the prior 2 years were not statistically significant (203 [10.3%] vs 183 [9.3%]; *P* = .06).

Table 3 shows ARRs and 95% CIs for receipt of influenza vaccination by April 1, 2024, for subgroups. Persons more likely to receive a vaccination than other groups included children, Asian individuals, and those who had received influenza vaccination in the past 2 years.

### Timely Vaccination by December 31, 2023 (Post Hoc)

Vaccination by December 31, 2023, was significantly higher in the video (3357 of 7410 [45.3%]; *P* = .007) and infographic (3288 of 7406 [44.4%]; *P* = .04) arms than in the control arm (3212 of 7417 [43.3%]) (eTable 1 in Supplement 2). Our multivariate analysis indicated that timely vaccination was higher in the video group (ARR, 1.05 [95% CI, 1.01-1.08]) and in the infographic group (ARR, 1.03 [95% CI, 1.00-1.07]) than in the control group (eTable 2 in Supplement 2). The number needed to treat to achieve 1 additional timely influenza vaccination for all ages was 50 in the video arm and 91 in the infographic arm.

### Influenza Vaccination Among Patients Who Opened Portal Messages (Post Hoc)

Within the video study arm, 2712 patients (36.6%) opened the portal questionnaire. Among them, 369 (13.6%) indicated that they watched the video, and 1626 (60.0%) received a vaccination by April 1, 2024. Within the infographic study arm, 3409 patients (46.0%) opened the portal questionnaire, and 1796 patients (52.7%) received a vaccination by April 1, 2024. We did not compare vaccine receipt within the subgroup that opened the portal messages to vaccine receipt for the control group since those who opened the portal messages may have greater predisposition to be vaccinated.

### Sensitivity Analyses

Sensitivity analyses were conducted using mixed-effects logistic regression and Poisson regression with physician fixed effects. The results were qualitatively consistent with the primary mixed-effects Poisson regression modeling approach.

Since we noted a preponderance of female physicians in our sample, we assessed the proportion of female physicians in the entire UCLA primary care population. We found that a greater proportion of active portal users were female (58%) and a higher proportion of patients attributed to participating health care clinicians were female (62%) than the proportion among all health care clinicians (58%).

### Primary Care Practitioner Survey Findings

**Figure 2.**
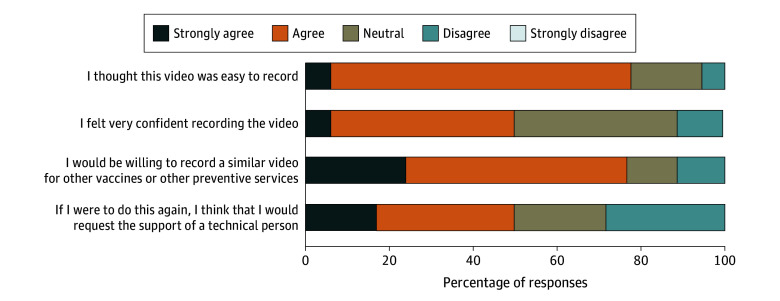
Primary Care Practitioner Responses Regarding the Feasibility and Acceptability of Making a Video Message About Influenza Vaccination

Of 21 physicians, 18 (85.7%) responded to the survey, with 10 recording the video on their phone, 4 on a computer, and 4 via video conferencing. The time to record the video (including practice and set-up) was less than 15 minutes for 3 physicians, 15 to 29 minutes for 7 physicians, 30 to 44 minutes for 4 physicians, 45 minutes to 1 hour for 1 physician, and longer than 1 hour for 3 physicians. Physicians rated the script as extremely helpful (n = 2), very helpful (n = 11), and somewhat helpful (n = 5); none reported that the script was slightly or not at all helpful. In total, 14 physicians received patient feedback. Of them, 4 indicated that the feedback was very positive, 8 that it was somewhat positive, and 2 that it was neither negative nor positive; none of the physicians received patient feedback that was somewhat or very negative. Figure 2 shows the findings regarding the feasibility and usability of creating an influenza vaccination video. Most physicians found the process easy and would record a similar video for other preventive services. Most physicians felt confident or neutral about recording the video, but many would request the support of a technical support person.

## Discussion

This RCT evaluated the effectiveness of patient portal–delivered physician messages—physician-created videos or a physician-specific infographic—on influenza vaccination rates for the primary outcome of end of season influenza vaccination for the entire UCLA Health study population. By the end of the vaccination season (primary outcome), neither the video nor the infographic was more effective than usual care. Among children assessed in the prespecified secondary analysis, both interventions were more effective: in the video intervention group, vaccination rates increased by 3.9 percentage points, and in the infographic group, by 0.6 percentage points.

This study was innovative by testing the effect of video and infographic messages sent by PCPs to their patients for a preventive measure. Our largely negative findings regarding end of season vaccination rates suggest that more intensive interventions are needed for the overall population. Such interventions may include training clinicians on vaccine communication (particularly regarding vaccine hesitancy)^40^ and office-based enhancements, such as health care clinician prompts, education, and standing orders.^41,42^

Our findings suggest the potential utility of a physician-created video for pediatric influenza vaccination. We do not know why the intervention was more effective for children than for adults. Given the single health system and modest number of children (n = 3200) assessed in this RCT, additional research is warranted.

Both interventions improved timely vaccination (post hoc analysis) before December 31 by a small amount for the overall population and a moderate amount for children. These interventions may be considered important because influenza often peaks in December or January in the US,^43^ and there is a lag of about 2 weeks between vaccination and immunity.^2^ Thus, vaccination by end of December is preferred.

### Strengths and Limitations

Study strengths include a pragmatic RCT design (to reflect typical clinical care settings), randomization within physicians’ patients to minimize unmeasured confounders, and high capture of influenza vaccination. Limitations include modest sample sizes for subgroups, inability to assess why patients did not receive a vaccine, and a single health system with a well-educated population having high access to vaccinations and likely predetermined views on influenza vaccination. Our only method to assess whether patients watched the video was through a question on the questionnaire (the portal mechanism to send patients a link to the video) about whether patients had watched the video; 36.6% of patients opened the questionnaire but only 13.6% of this subgroup responded to the questionnaire that they had watched the video. We suspect that many more patients watched the video but did not respond to the portal questionnaire. Also, interventions were in English (<2% of the population requests another language), and the eligible population was portal users (>90% of the UCLA Health population). In addition, our study had a higher proportion of female participants than that occurring in the UCLA primary care population.

Additionally, some patients may have received vaccinations from external settings that did not link with either the California Immunization Information System (CAIR) or Surescripts; others may have opted out of allowing pharmacy-administered data to be shared with CAIR. However, these would be balanced across study arms.

## Conclusions

In this RCT testing the effect of 2 types of patient reminders, neither portal-delivered video messages about influenza vaccination personalized by PCPs nor infographics showing the physician’s photograph increased influenza vaccinations by the end of the influenza season for the entire UCLA Health patient study population compared with usual care. However, both interventions were effective for children. Video messages appeared to increase timely influenza vaccination (by December 31) for the entire population and particularly among children (by April 1, 2024, and by December 31, 2023). Video messages, personalized by PCPs, may represent another potential strategy to improve influenza vaccination for children and perhaps to improve timely influenza vaccination across the age spectrum.

## Notes

Supplement 1.
Trial Protocol

Supplement 2. **eAppendix 1.** Examples of Patient Portal Messages **eAppendix 2.** Instructions and Scripts for Physician Video Messages **eAppendix 3.** Example of Infographics **eAppendix 4.** Provider Follow-Up Survey Questions **eTable 1.** Influenza Vaccination Rates by Condition and Subgroups by December 31, 2023 **eTable 2.** Adjusted risk Ratios (95% Cis) for Influenza Vaccination by December 31, 2023, by Study Group and Patient Characteristics, Using Mixed Effects Poisson Model (Clustering by Provider) of Vaccination Status

Supplement 3.
Data Sharing Statement
