# Supplementary material for: Video and Infographic Messages From Primary Care Physicians and Influenza Vaccination Rates: A Randomized Clinical Trial
Source: JAMA Netw Open. 2025 Aug 13;8(8):e2526514. doi: 10.1001/jamanetworkopen.2025.26514 (PMC12351418; doi:10.1001/jamanetworkopen.2025.26514)
Supplement: Supplement 1. — Trial Protocol [file jamanetwopen-e2526514-s001.pdf]

ID: IRB#17-001889
Principle Investigator: Peter G Szilagyi
Date: 9/29/23
Title: *Improving Influenza Vaccination Delivery Across a Health System by the Electronic Health Records Patient Portal*

Submission Date: 12/14/2017 **IRB Approval Date:** 12/20/2017
RCT Amendment Approval Date: 8/11/2023 **Provider Survey Amendment Approval Date:** 7/22/24

Research Design and Methods:
Describe in detail the design and methodology of the study.

*The randomized clinical trial described below is part of research study which covers six years, and six distinct RCTs. The full IRB application, "*UCLA IRB Snapshot- Protocol and SAP – Approved 7.22.24", *includes the description of all the previous trials conducted from 2017-2023. Below is only the description of the RCT #6 conducted 2023-2024 and accompanying provider surveys; this manuscript reflects RCT #6.*

SPECIFIC AIMS

The overall purpose of the RCTs is to evaluate the impact that R/R, delivered through the patient portal, could have on the flu immunization rates of UCLA Health patients. The intent is to ultimately take the information learned and create and disseminate an adaptable toolkit to other health systems.

Specific aims are:

Aim #1: Adapt algorithms, educational messages, and protocols previously used for mailed or phone influenza vaccine R/R, to create a patient portal research platform. (Aim 1 is outlined in detail in IRB applications titled: Patient Portal - Patient Qualitative Interviews and Patient- Portal Provider Qualitative Interview)

Aim #2: Assess the impact of portal R/R and key design features upon flu vaccination rates and costs.

> 2h. RCT #6: Using a 3-arm trial with a standard-of-care control group (up to 27 practices). Compare effectiveness of sending portal-based video from patient's PCP vs. sending portal-based image on influenza vaccination rates vs. standard-of-care group.
>
> RCT #6 will test:
> a. The main effect of portal-based PCP video message- v. portal-based image message on flu vaccination rates

OVERALL STUDY DESIGN

Subjects receiving the intervention will be patients at one of the primary care practices within the UCLA Health System (up to 65 practices), and will include patients of any age. A proportion of the patients from these practices will be selected to participate - i.e. those who meet the inclusion criteria. Randomization techniques will occur before each RCT.

For RCT 6, a smaller subset of primary care practices will be included (up to 27 practices), only 1 PCP from each practice will record videos (up to 27 PCPs), only subjects who see a participating PCP will be included in the RCT but must still meet the below inclusion criteria. Subjects of each PCP will be randomized into 1 of 3 groups: 1) To receive the portal-based PCP video messages (from their own PCP), or 2) to receive the portal-based image message, or 3) to receive standard-of-care messages.

Number of Subjects

In RCT #6 there will be up to 30,000 primary care patients. In RCT 6, if multiple members of a household see the same PCP, a single patient will be randomly selected per household for inclusion in the analysis, the rest of the household members will be excluded from the study to minimize confusion. Creation of family units is described in the methods below.

Gender and age: We anticipate the gender distribution to be roughly equal. All ages of patients will be included in the study; however, patient will not receive a message until they are 6 months of age, as the flu vaccine is not recommended for those less than 6 months of age.

Racial and ethnic origin: There are no enrollment restrictions based on race/ethnicity.

Inclusion criteria

RCT 6: For RCT 6, a smaller subset of primary care practices will be included (up to 27 practices), only 1 PCP from each practice will record videos (up to 27 PCPs), only subjects who see a participating PCP will be included in the RCT. Participating PCPs will record video messages explaining the importance of the flu vaccine and urging their patients to get it. An infographic image will also be used which highlights the importance and benefit of the influenza vaccine which will be sent via the portal.

A patient at one of the participating UCLA primary care providers (up to 27 providers from 27 practices). An individual is deemed a primary care patient of the UCLA Health System through the following algorithm: Assigned managed care patients (UCLAMG) + Attributed patients from other payers/ACOs

1. =2 PCP visits in the past 3 years; or
2. =1 PCP visit with preventive service code in past 1 year (99381-99397 or G0438/G0439)
3. All visits cannot be urgent care visits (ie excludes visits after hours or on weekends, not by urgent care codes since UCLA does not bill accordingly)

Active patient: We decided to use the algorithm outlined above and currently approved and in place by the UCLA Health System as the research team believes it to be a generalizable model that could be applied to other health systems.

Exclusion criteria

Patients will be excluded from the overall study if they are not part of UCLA's primary care registry per the above algorithm detailed in the inclusion criteria.

Creating family units

An overall address field will be constructed from the data pull including the address, city, state, and zip code fields. Primary telephone number, patient ID of the guarantor and insurance member ID will be used as other variables in the process to create the family units. The following steps taken to create these family units is described below:

1. Start with a single entry in the contact data pull
2. Add any other entries with the same patient ID as any existing entry to the family
3. Add any other entries with the same address as any existing entry to the family
4. Add any other entries with the same primary phone as any existing entry to the family
5. Add any other entries with a patient ID matching the patient ID of guarantor as any existing entry to the family
6. Add any other entries with the same insurance member ID as any existing entry to the family
7. Repeat steps 2-6 until no new matches are found - this forms a single family
8. Repeat steps 1-7 on the remaining entries to build each additional family until all entries have been associated with a family.

Please note - when there were errors with the address and telephone number, these variables were not used to group families (ex. instances with 20+ entries with the same data for telephone number).

In RCT 6, the same process will be used for grouping families, however, MRN of the guarantor and contact information for the guarantor, as well as Patient ID, Patient MRN and contact information of the emergency contact will be added to enhance our ability to identify possible family units.

Definition of active portal user:
We can only send portal R/R messages to patients who have signed up for the portal. Since some patients sign up for the portal but never use it, we define an active portal user as a patient (or proxy on behalf of the patient) who has used the portal within 12m (~59% of UCLA patients), and who has logged in 1 or more times in the past 12months (reference date for login activity will be selected by the research team and then working backwards by 365days, example of a range 8/1/22 - 7/31/23 for RCT #6 excluding their initial activation login and any subsequent logins on the same date of account activation.]

Selection of index patient from each family unit
1. If the family contains any active portal user per our definition above, the index patient is randomly selected from among these patients.
2. If the family contains no active portal users, the index patient is randomly selected from all patients in the family.

Selection of the index patient for primary analysis:
1. An active portal user per the definition above
2. The patient is affiliated with one of the primary care clinics of interest
3. The patient was randomly selected as the index patient in their family unit

Only active users affiliated with included UCLA primary care practices will be included in the study sample. We will assess primary and secondary intervention effects among eligible index patients, and the remainder of the household will not be included in the study.

METHODS AND STUDY PROCEDURES
The UCLA Health System is made up in part of approx 65 primary care practices. The leadership team at UCLA Health has agreed to allow these practices to participate in the 6 RCTs. The full description of RCTs 1-5 can be found under “UCLA IRB Snapshot- Protocol and SAP – Approved 7.22.24”. RCT 6 is outlined below (conducted 2023-24).

Randomization: We plan a 3-arm trial with a standard-of-care control group. We will randomize patients within PCP patient lists to (Arm 1) portal-based PCP video message versus (Arm 2) portal-based image message v. (Arm 3) control. Three monthly reminders will be sent in early October, late October (due to an anticipated CareConnect upgrade in November) and December.

Arm 1: Patients randomized to this arm will first receive an email-based "tickler message" alerting them to a video-message in their portal and providing them with a link to guide them to the portal (see "RCT 6_Intervention Groups_Tickler messages_Questionnaires" for content). In the portal they will see a video message recorded by their own PCP explaining the importance and benefits of receiving the influenza vaccine (see 'RCT 6_Video Intervention_Physician Script' for message content). The video message will have an accompanying questionnaire which will ask the patients if they watched the video, and questions about if and where they plan to get the influenza vaccine (see "RCT 6_Intervention Groups_Tickler messages_Questionnaires" for content).

Arm 2: Patients randomized to this arm will first receive an email-based "tickler message" alerting them to a message in their portal and providing them with a link to guide them to the portal (see "RCT 6_Intervention Groups_Tickler messages_Questionnaires" for content). In the portal they will see an infographic with their own PCP's name and image on it. The infographic will explain the importance and benefits of receiving the influenza vaccine (see "RCT 6_Image Intervention" for content). The image message will have an

accompanying questionnaire which will ask the patients questions about if and where they plan to get the influenza vaccine (see "RCT 6_Intervention Groups_Tickler messages_Questionnaires" for content).

Arm 3: Patients randomized to this arm will receive standard-of-care UCLA messages

We again will select 1 individual per household. We will use a subset of 27 UCLA-based primary care practices (n=up to 27 practices, n = up to 30,000 patients).

For the RCT 6 provider survey we will send all 21 providers who recorded videos a survey via REDCap (see "RCT 6 Provider Survey Questions") . The REDCap survey will include a section asking for the providers name, which will allow us to track the completion of provider surveys. We will send up to 5 reminder messages via email to non-responders.

MEASURES

The key independent variables are the portal video message v. the portal image message. The study arms will be measured across two study populations (pediatric 6 mo-18 y and adult 18<).

STATISTICS AND ANALYTIC PLAN

Analytic Plan:

The primary outcome will be the patient's end of flu season vaccination status. Intervention effects will be assessed using mixed effects log-binomial models. Models will contain terms for messaging type (video v. image). Models will adjust for patient characteristics, including age, sex, race/ethnicity, primary language, primary insurer, and prior year vaccination status. Practice random effects will be used to account for clustering of patients by primary care practice and will involve performing model contrasts, and a significance level of 0.017 will be used (3-fold Bonferroni correction of 0.05). These analyses will use a significance level of 0.05. Intervention effects will be summarized in terms of risk ratios and 95% confidence intervals. Exploratory analyses will evaluate treatment effect heterogeneity by patient characteristics. In addition, non-portal users will be excluded from the final analyses.

Power Analysis:

Power was approximated using a chi-squared test comparing each combined intervention arm (1/3rd of included patients) with the standard of care control arm (1/3rd of included patients). This is a conservative simplification of the planned mixed effects log binomial regression analysis. The planned design provides >90% power to detect a minimally clinically significant 2 percentage point difference in vaccination rates. This conservatively assumes a control arm vaccination rate of 50% (for maximum variability), and an alpha of 0.017.
