# Supplementary material for: Video and Infographic Messages From Primary Care Physicians and Influenza Vaccination Rates: A Randomized Clinical Trial
Source: JAMA Netw Open. 2025 Aug 13;8(8):e2526514. doi: 10.1001/jamanetworkopen.2025.26514 (PMC12351418; doi:10.1001/jamanetworkopen.2025.26514)
Supplement: Supplement 2. — eAppendix 1. Examples of Patient Portal Messages eAppendix 2. Instructions and Scripts for Physician Video Messages eAppendix 3. Example of Infographics eAppendix 4. Provider Follow-Up Survey Questions eTable 1. Influenza Vaccination Rates by Condition and Subgroups by December 31, 2023 eTable 2. Adjusted risk Ratios (95% Cis) for Influenza Vaccination by December 31, 2023, by Study Group and Patient Characteristics, Using Mixed Effects Poisson Model (Clustering by Provider) of Vaccination Status [file jamanetwopen-e2526514-s002.pdf]

Supplementary Online Content

Szilagyi PG, Clark EJ, Duru OK, et al. Video and infographic messages from primary care physicians and influenza vaccination rates: a randomized clinical trial. *JAMA Netw Open*. 2025;8(8):e2526514. doi:10.1001/jamanetworkopen.2025.26514

eAppendix 1. Examples of Patient Portal Messages

eAppendix 2. Instructions and Scripts for Physician Video Messages

eAppendix 3. Example of Infographics

eAppendix 4. Provider Follow-Up Survey Questions

eTable 1. Influenza Vaccination Rates by Condition and Subgroups by December 31, 2023

eTable 2. Adjusted risk Ratios (95% Cis) for Influenza Vaccination by December 31, 2023, by Study Group and Patient Characteristics, Using Mixed Effects Poisson Model (Clustering by Provider) of Vaccination Status

This supplementary material has been provided by the authors to give readers additional information about their work.

eAppendix 1. Examples of Patient Portal Messages

Portal Video Message Intervention Tickler Message:

(Same message used for patients of internal, pediatric, med-peds, family medicine providers)

Subject line: Dr. (PCP name) has a message for you

Hello [Patients preferred name],<br><br>I have recorded a **short video message** for you about your health. Please click the link below to see my brief video in myUCLAhealth.<br><br>**[Click here for Dr. (PCP)'s message to you]**<br><br>Thank you,<br>[Primary Care Provider's name]

Portal Infographic Message Intervention Tickler Message:

(Same message used for patients of internal, pediatric, med-peds, family medicine providers)

Subject line: Dr. (PCP name) has a message for you

Hello [Patients preferred name],<br><br>I have a **message** for you about your health. Please click the link below to see my brief message in myUCLAhealth.<br><br>**[Click here for Dr. (PCP)'s message to you]**<br><br>Thank you,<br>[Primary Care Provider's name]

© 2025 Szilagyi PG et al. *JAMA Network Open*.

Portal Video Message Intervention Questionnaire:

Subject line: Video message from [PCP name] regarding your flu vaccine

Message Body:

[Patients preferred name], I recorded this video for you. Click here to watch:

[VIDEO]

1. Did you watch my video?
   Yes/No

[Questions below will stay hidden until patients answer question 1]

2. How important do you think it is to get the flu vaccine this year?
   Very important/Important/Somewhat important/Not important

3. Where will you get the flu vaccine this season?
   a. UCLA Health
   b. Pharmacy
   c. School/Workplace
   d. Undecided/Other
   e. I already got the flu vaccine this season
   f. I don't plan on getting a flu vaccine this season

If you would like to get your vaccine at UCLA Health, **click here** to schedule your vaccine appointment.

Complete/Finish later/Cancel

© 2025 Szilagyi PG et al. *JAMA Network Open*.

Portal Infographic Message Intervention Questionnaire:

Subject line: Message from [PCP name] regarding your flu vaccine

Message body:

[INFOGRAPHIC]

1. How important do you think it is for you to get the flu vaccine this year? Very important/Important/Somewhat important/Not important

2. Where will you get your flu vaccine this season?
   a. UCLA Health
   a. Pharmacy
   b. School/Workplace
   c. Undecided/Other
   d. I already got the flu vaccine this season
   e. I don't plan on getting a flu vaccine this season

If you would like to get your vaccine at UCLA Health, **click here** to schedule your vaccine appointment.

Complete/Finish later/Cancel

Portal Video Intervention 'myUCLAhealth' Message:

(Found in 'automated messages' section of patient's myUCLAhealth account and served as a backup route to access the Questionnaire with the video if patients did not follow the tickler message link)

Subject line: Your primary care doctor has a video message for you

Hello [Patients preferred name],

I have recorded a **short video message** for you about your health. Please click the link below to see my brief video in myUCLAhealth.

[Video message from [PCP name] regarding your flu vaccine]

© 2025 Szilagyi PG et al. *JAMA Network Open*.

Portal Infographic Intervention 'myUCLAhealth' Message:

(Found in 'automated messages' section of patient's myUCLAhealth account and served as a backup route to access the Questionnaire with the infographic if patients did not follow the tickler message link)

Subject line: Your primary care doctor has a video message for you

Hello [Patients preferred name],<br><br>I have recorded a **message** for you about your health. Please click the link below to see my brief video in myUCLAhealth.<br><br>**[Message from [PCP name] regarding your flu vaccine]**

© 2025 Szilagyi PG et al. *JAMA Network Open*.

eAppendix 2. Instructions and Scripts for Physician Video Messages

Instructions for video recordings for all providers (Internal/Med-Peds/Family/Peds):

INSTRUCTIONS:

- Please create a short video, **<60 seconds**.
- Tone: We are hoping for a **caring and upbeat tone**, nothing designed to inspire fear in our patients or to overwhelm them with statistics.
    - Please try to speak in your authentic voice
    - The key is that we want you to **come across with directness and warmth**, as if you were talking to someone sitting in front of you.
        - Please look directly at the camera
- Content: ***please get your flu vaccine this year***
    - Below is a sample script as well as some key points you may want to use.
    - If you prefer to ad lib, that's fine, too.
    - While we are asking you to stick to much of the content, please use your own words
- A sample video is available at xxx

In general, it is considered that most people speak at a rate of around 110-150 words per minute in normal conversation, but it's worth mentioning that some people can speak as fast as 250 words per minute in certain situations, like when they are reading a script or reading aloud a passage.

KEY POINTS you may want to include:

1. I care: I'm asking you to get a flu vaccine this year because I care about you.
    - I'm creating this video because this is an issue that is important to me because I care about your health.
    - I really care about your health
    - I'm telling you what I would tell my mom or family member

1. The main reason I want you to get the flu vaccine this fall is because **it will help you stay healthier**. Without the flu, you can keep up with your responsibilities and your pleasures.
2. Like me, you may want to get the flu vaccine because you want to **reduce the chance of getting those around you sick**. I get the flu vaccine for my own well-being, but also because I don't want to spread flu to my loved ones, my patients, and my co-workers!
3. The **flu vaccine is safe**. It may rev up your immune system so you feel like you are coming down with something; if so, just take ibuprofen or acetaminophen the next day.
4. We don't know when the flu virus will arrive in southern California, so it's better to get the flu vaccine **in the fall** rather than waiting until we hear about flu outbreaks.
5. So, I'm asking you to pull out your calendar now and **schedule your flu vaccination date**. Whether you come to our office or get it at your nearest pharmacy – whatever works for you. My point is that I want you to schedule it now so you will get it done soon.
6. **Thanks for listening** to me & trusting me with your care.

Other points:

- "Most of my patients get the flu shot"
- "I and my family get the flu shot every year"; "I get my flu shot every year and my family gets it every year.
- "While elder people and young children are at higher risk, I recommend this vaccine to all my patients."
- You're not going to miss work and your kids won't miss school

© 2025 Szilagyi PG et al. *JAMA Network Open*.

Internal/Med-Peds/Family Medicine Video Sample Script:

Hi. I am reaching out to urge you to get a flu vaccine soon because I care about you. I'm telling you what I tell my own family: the flu vaccine will help you avoid the flu so you can keep up with your work, family, and fun activities. I get the flu vaccine for my own health and because I don't want my loved ones or my patients to get sick! I'm recommending it to you because I'm confident it is a safe and effective vaccine.

Please do me a favor by scheduling your flu vaccination right now and marking it in on your calendar. You can get it from my office by calling us or by scheduling through myUCLAhealth. You could also get it at your pharmacy. Wherever you decide to get it, the key is to schedule it now so that you'll be protected as soon as possible. Please let me know your plans by answering 3 quick questions right after this video.

Thanks for listening to me and trusting me with your care.

Pediatrics Video Sample Script:

Hi. I am reaching out to urge you to get your child a flu vaccine soon because I care about your child and family. I'm telling you what I tell my own family: the flu vaccine will help your child avoid the flu so they can keep up with their school, family, and fun activities. I get the flu vaccine for my own health and because I don't want my loved ones or my patients to get sick! I'm recommending it to your child because I'm confident it is a safe and effective vaccine for all ages.

Please do me a favor by scheduling your child's flu vaccination right now and marking it in on your calendar. You can get it from my office by calling us or by scheduling through myUCLAhealth. You could also get it at your pharmacy. Wherever you decide to get it, the key is to schedule it now so that your child will be protected as soon as possible. Please let me know your plans by answering 3 quick questions right after this video.

Thanks for listening to me and trusting me with your child's care.

© 2025 Szilagyi PG et al. *JAMA Network Open*.

eAppendix 3. Example of Infographics

Internal/Med-Peds/Family Medicine Infographic sent via portal message (photo with permission)

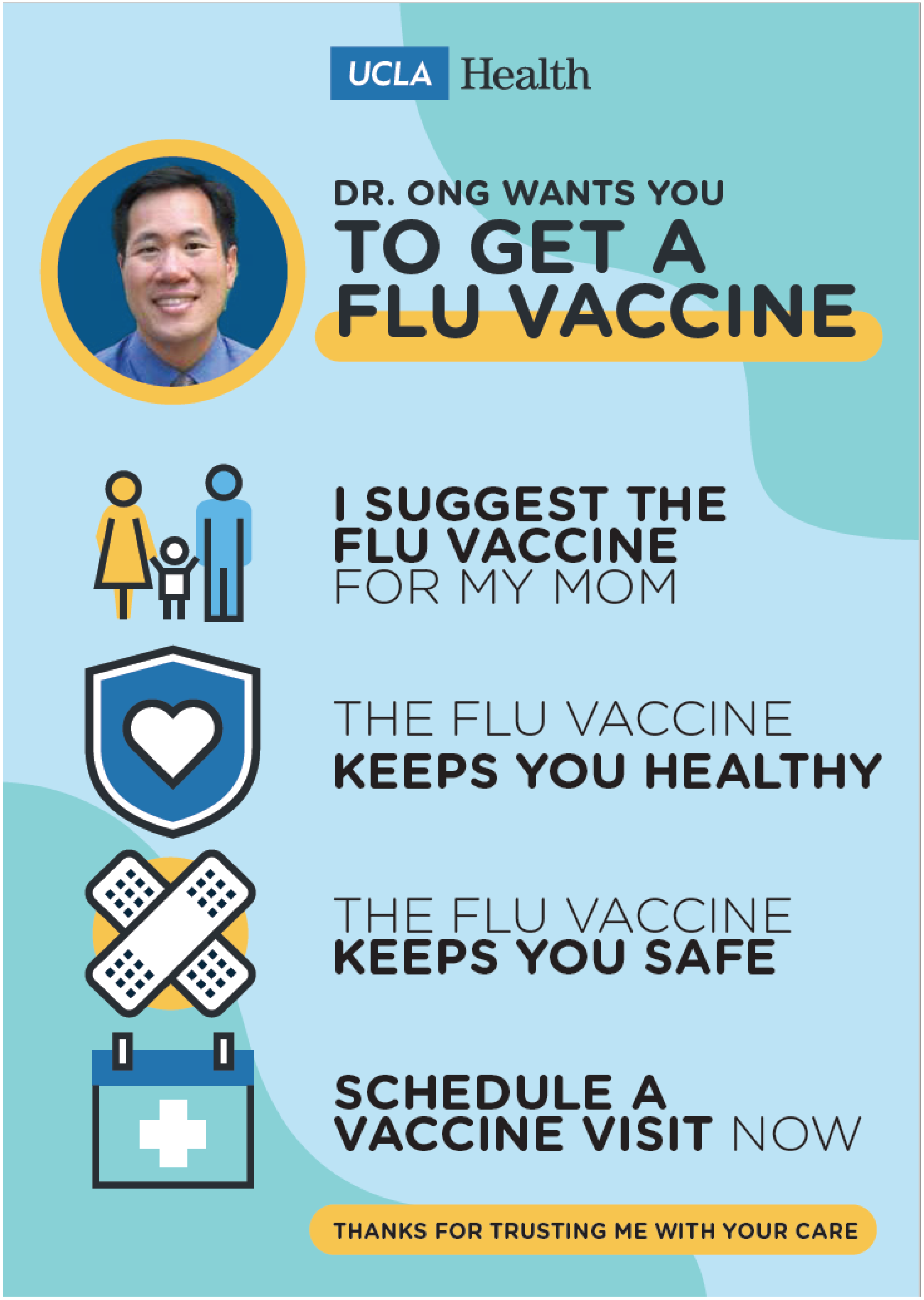

© 2025 Szilagyi PG et al. *JAMA Network Open*.

Pediatrics Infographic sent via portal message (photo with permission)

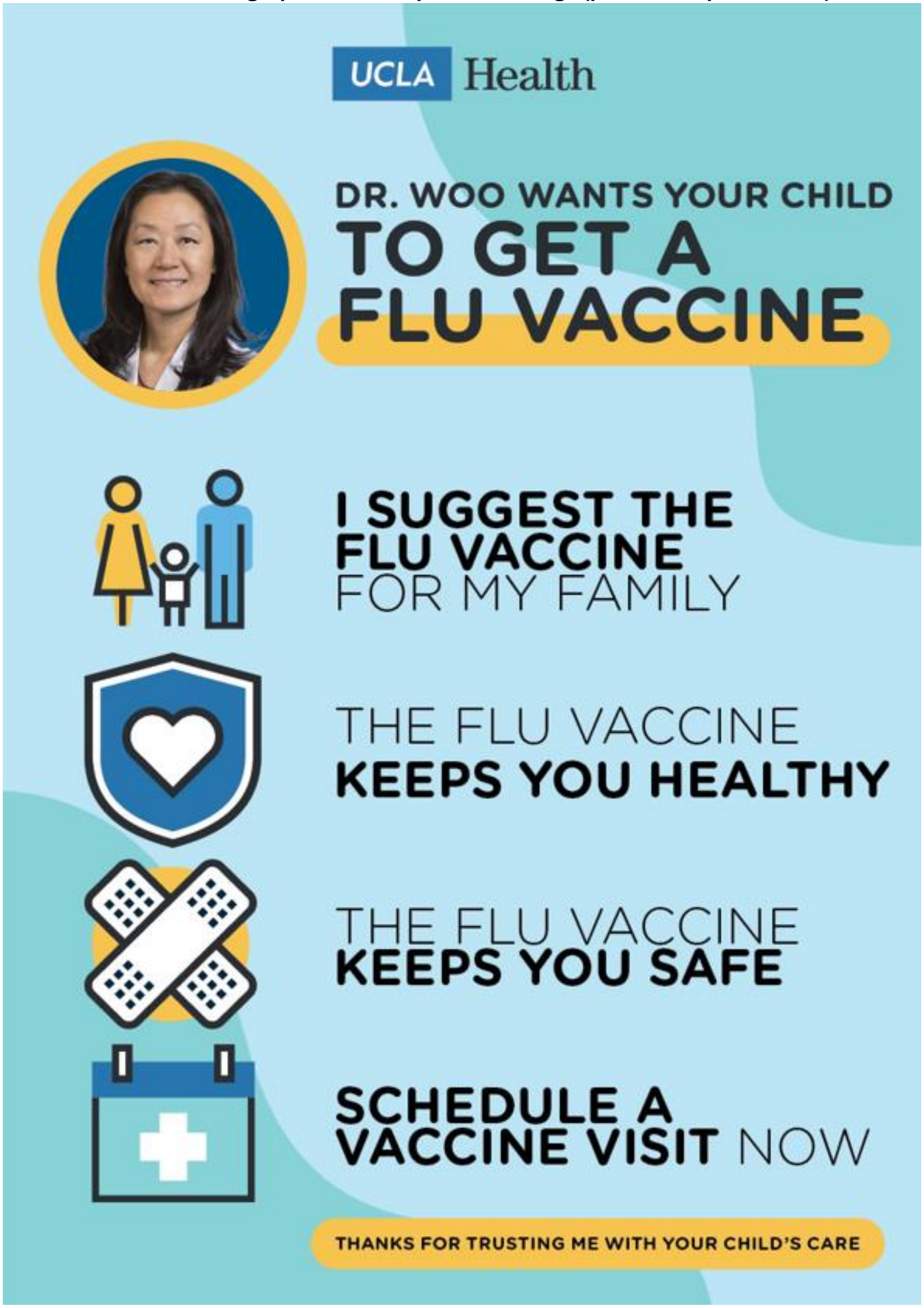

© 2025 Szilagyi PG et al. *JAMA Network Open*.

eAppendix 4. Provider Follow-up Survey Questions

1. **Do you agree to participate in this research survey?**
    a. Yes
    b. No
2. **How did you record your video?**
    a. On my phone
    b. On my computer/laptop
    c. Via Zoom with research team
    d. Other, please specify:

3. **How long did it take you to record your video (including if you took time to practice, set up, etc.)**
    a. Less than 15 minutes
    b. 15-29 minutes
    c. 30-44 minutes
    d. 45 minutes-1 hour
    e. More than 1 hour

4. ***I thought the video was easy to record.**
    a. Strongly disagree
    b. Disagree
    c. Neutral
    d. Agree
    e. Strongly agree

5. ***I felt very confident recording the video.**
    a. Strongly disagree
    b. Disagree
    c. Neutral
    d. Agree
    e. Strongly agree

6. ***I would be willing to record a similar video for other vaccines or other preventive services.**
    a. Strongly disagree
    b. Disagree
    c. Neutral
    d. Agree
    e. Strongly agree

7. ***If I were to do this again, I think that I would request the support of a technical person.**
    a. Strongly disagree
    b. Disagree
    c. Neutral

© 2025 Szilagyi PG et al. *JAMA Network Open*.

   d. Agree
   e. Strongly agree

8. **How helpful was it to have a basic script provided to you?**
   a. Not at all helpful
   b. Slightly helpful
   c. Somewhat helpful
   d. Very helpful
   e. Extremely helpful

9. **Did you hear any feedback about your video from your patients?**
   a. No
   b. Yes
      (If selected) **On average the comments were:**
      i. Very negative
      ii. Somewhat negative
      iii. Neither negative nor positive
      iv. Somewhat positive
      v. Very positive

10. **Additional comments:** [Free text]

© 2025 Szilagyi PG et al. *JAMA Network Open*.

eTable 1. Influenza Vaccination Rates by Condition and Subgroups by December 31, 2023

Vaccination Rates by Study Condition: No. (%) Vaccinated by December 31, 2023					
		Video Messages		Infographic Message	
**Patient Characteristic**	**Control**	**No. (%)**	**P-Values**	**No. (%)**	**P-Values**
**All Patients**	**3212 (43.3)**	**3357 (45.3)**	**<0.01**	**3288 (44.4)**	**<0.05**
**Age (years)**					
**0.5 (6 mos) to <18**	511 (48.3)	569 (53.8)	<0.001	544 (50.1)	<0.05
**18-49**	1144 (34.2)	1241 (36.9)	<0.05	1209 (35.4)	
**50-64**	646 (42.0)	669 (43.3)		663 (44.2)	
**65+**	913 (61.7)	879 (60.9)		876 (62.3)	
**Gender**					
**Women**	2040 (43.7)	2100 (45.2)	<0.001	2068 (44.4)	
**Men**	1171 (42.6)	1257 (45.5)		1220 (44.4)	<0.1
**Primary Insurer**					
**Private**	2564 (40.9)	2750 (43.6)	<0.001	2691 (42.7)	<0.01
**Public**	601 (58.1)	556 (55.8)		553 (55.5)	
**Other/Unknown**	47 (41.6)	52 (48.6)		46 (42.6)	
**Race**					
**White**	1647 (45.3)	1864 (49.1)	<0.001	1740 (48.1)	<0.05
**Black**	97 (28.7)	92 (28.2)		94 (26.7)	
**Asian**	484 (57.4)	433 (55.8)		452 (56.9)	
**Other (Multiple or Unknown)** [a]	983 (37.8)	969 (38.6)		41 (38.1)	
**Ethnicity**					
**Hispanic**	1044 (36.9)	391 (38.3)		368 (36.5)	
**Non-Hispanic/Unknown**	6373 (44.4)	6390 (46.4)	<0.01	2924 (45.7)	<0.05
**Influenza Vaccine History (Past 2y)**					
**None**	161 (8.2)	181 (9.3)	<0.1	176 (8.9)	
**Influenza vaccination**	3054 (56.0)	3183 (58.2)	<0.05	3113 (57.3)	<0.05

© 2025 Szilagyi PG et al. *JAMA Network Open.*

eTable 2. Adjusted risk Ratios (95% Cis) for Influenza Vaccination by December 31, 2023, by Study Group and Patient Characteristics, Using Mixed Effects Poisson Model (Clustering by Provider) of Vaccination Status

Adjusted Risk Ratios (95% CIs) for Vaccination by December 31, 2023	
**Group**	**Adjusted RR (95% CI)**
**Modality (Ref = Control)**	
**Video**	1.05 (1.01-1.08)
**Infographic**	1.03 (1.00-1.07)
**Age (Ref = <18)**	
**18-49**	0.75 (0.67-0.83)
**50-64**	0.89 (0.80-0.99)
**65+**	1.12 (1.01-1.25)
**Male (Ref = Female)**	1.01 (0.98-1.04)
**Primary Insurer (Ref = Private)**	
**Public**	1.01 (0.96-1.06)
**Other/Unknown**	0.99 (0.87-1.14)
**Race (Ref = White)**	
**Black**	0.77 (0.70-0.86)
**Asian**	1.14 (1.10-1.18)
**Other (Multiple or Unknown)°**	0.90 (0.88-0.93)
**Hispanic (Ref = Non-Hispanic/Unknown)**	0.93 (0.87-0.99)
**Influenza Vaccine History (Past 2y) (Ref = No)**	5.97 (4.25-8.39)

© 2025 Szilagyi PG et al. *JAMA Network Open*.
