# Supplementary material for: Video and Infographic Messages From Primary Care Physicians and Influenza Vaccination Rates: A Randomized Clinical Trial
Source: JAMA Netw Open. 2025 Aug 13;8(8):e2526514. doi: 10.1001/jamanetworkopen.2025.26514 (PMC12351418; doi:10.1001/jamanetworkopen.2025.26514)
Supplement: Supplement 3. — Data Sharing Statement [file jamanetwopen-e2526514-s003.pdf]

Data Sharing Statement

Szilagyi. Video and Infographic Messages From Primary Care Physicians and Influenza Vaccination Rates. *JAMA Netw Open*. Published August 13, 2025.
doi:10.1001/jamanetworkopen.2025.26514

Data
Additional Information: ClinicalTrials.gov identifier: NCT06062264
Data available: Yes
Data types: Deidentified participant data
How to access data: Please contact the author for requests for data:
pszilagyi@mednet.ucla.edu
When available: With publication

Supporting Documents
Document types: None

Additional Information
Who can access the data: Anyone requesting the data
Types of analyses: For a specified purpose
Mechanisms of data availability: With investigator support and with a signed data access agreement
